# Novel lung imaging techniques: new experimental insights in acute respiratory failure

**DOI:** 10.1186/s40635-024-00711-x

**Published:** 2025-01-17

**Authors:** Mariangela Pellegrini, Maurizio Cereda

**Affiliations:** 1 Anesthesia, Operation and Intensive Care Medicine, Uppsala University Hospital, Uppsala, Sweden; 2 Hedenstierna Laboratory, Department of Surgical Sciences, Uppsala University, Akademiska Sjukhuset Ing 40 3 Tr, 75185 Uppsala, Sweden; 3 Department of Anesthesia, Critical Care and Pain Medicine, Massachusetts General Hospital, Boston, USA; 4 Harvard Medical School, Boston, USA

Quantitative lung imaging entails the derivation of relevant physiological—rather than diagnostic—information from radiological data sets. This approach significantly contributed to elucidating pathophysiology and guiding ventilatory management of acute respiratory failure [1]. Thanks to its ability to visualize the lungs in three dimensions and dynamically thought time, computed tomography (CT) demonstrated damaging patterns of inflation such as excessive stretch of residual ventilated airspaces (‘baby lung’) and intermittent airway collapse during mechanical ventilation. More recently, sophisticated CT image analysis techniques and positron emission tomography (PET) allowed the identification of regions of the lung at risk of worsened injury from the ventilator [2, 3]. Such ability to investigate lung behaviour at a regional level is essential for optimising mechanical ventilation strategies.

Advanced techniques, such as positron emission tomography (PET), synchrotron radiation scanning, and magnetic resonance imaging (MRI), generate novel insights into the mechanisms of lung injury. Although widely used, each imaging technique has a high potential to evolve and improve, always addressing new methodological approaches. For example, improved techniques of perfusion imaging may reveal the role of abnormal distribution of pulmonary blood flow and ventilation/perfusion mismatch in the severity and progression of lung injury [4].

The risk of transporting critically ill patients and the uncertain integration of radiological data into clinical management limit the use of quantitative imaging techniques in acute respiratory failure. Nevertheless, improved bedside techniques (e.g., electrical impedance tomography and lung ultrasonography) and developments in three-dimensional imaging, including low-dose and bedside CT scanning, can potentially address the increasing interest in delivering personalised mechanical ventilation.

Furthermore, artificial intelligence is transforming image analysis, streamlining and accelerating quantitative image analysis (segmentation, registration) and patient classification (identification of injury patterns) to predict outcomes and treatment responses in the critically ill [5]. Combining robust data analysis methods and lung imaging further increases the potential benefit in intensive care.

Although the fundamental principles of physiology remain constant, advances in lung imaging technologies have granted us new perspectives—“a new set of eyes”—to explore respiratory physiology in ways never before possible. These technological advancements allow for unprecedented accuracy and detail in assessing physiological processes and pathophysiological mechanisms within the lung. By integrating advanced imaging modalities with cutting-edge data analysis, researchers can uncover intricate relationships between ventilation, perfusion, and lung mechanics, providing previously inaccessible insights. This transformation enables a deeper understanding of the complex physiological underpinnings of acute respiratory failure and opens new opportunities for targeted interventions and personalised care.

This thematic collection will focus on new experimental insights, physiological understanding, and monitoring of acute respiratory failure provided by the surge in novel lung imaging technologies. We invite authors to contribute with experimental studies, with both translational and clinical insights, employing imaging techniques to explore and expand our knowledge of respiratory physiology and the pathophysiological mechanisms underlying acute respiratory failure in critical care settings.

This collection aims to serve as a foundation resource, highlighting how modern imaging technologies are reshaping our understanding of respiratory physiology and driving progress in the management of critically ill patients.

## Data Availability

Not applicable.
